# Science and the Prevention and Cure of Disease

**Published:** 1918-10-12

**Authors:** 


### SCIENCE AND THE PREVENTION AND CURE OF DISEASE.

The prevention of disease is an ideal which has inspired much scientific investigation, much legislative enterprise, and much administrative activity, and though the goal is still far ahead the struggle towards it may boast not a few practical triumphs. Some of these triumphs have been written so plainly by the history of the war that their value receives universal recognition. Not generally known, however, is it that the basis of the practices which have so largely preserved our armies from epidemic and other diseases has been provided by exact scientific work, much of which was conducted in hospitals and laboratories far from the scene of the fighting and in days when war was only a terrible possibility. It is because, by investigations of this order, the causes of certain diseases were made manifest that prevention of the corresponding maladies became possible. Here, as elsewhere, knowledge has justified itself as power, and the lesson is an obvious one. Clearly our scientific workers must be encouraged and helped to still further tasks, and their work must be recognised as an essential element in an intelligent and ordered war against disease. Yet it is necessary to deal with the actual realities of the situation. Whether or not the time will ever come when the scourge of disease will be completely banished from the experience of mankind it is certain that such an achievement is not yet. Hence there remains the problem of the care of individual sufferers, and though this may be classed as a much smaller ambition it is none the less an urgent and practical need. Is it possible that the scientific work which has done so much for the prevention of disease may also do something for its cure?

Such, in substance, is one of the questions raised by Dr. C. H. Browning in the address he recently delivered at the opening of the medical session at the Middlesex Hospital. Dr. Browning recognised that prevention is not always possible, and that for generations yet to come efforts at cure will form a necessary and worthy enterprise. In his belief severe and organised scientific work may lead to the discovery and preparation of remedial agents of trustworthy therapeutic value, and he urges that such work should be undertaken. Naturally, as he himself is a director of a pathological laboratory, he looks for success mainly from laboratory workers, though he recognises that clinical observations and tests must be included in the scheme. What he advocates is a "massed attack" on disease with the organised co-operation of all scientific and clinical resources. The claim seems to us an appropriate one. Prevention is so attractive an aim that the care of the individual patient is in some danger of being obscured by it. Yet the latter, as well as the former, is a function of practical medicine, and is certain to continue to present itself for many a day. Doubtless a good deal of drug administration is arbitrary and haphazard and without scientific knowledge. But pharmacology has made some advances, and Dr. Browning's plan for its further pursuit has abundant justification. What must go along with it for anything like full success is a capacity to detect the very beginnings of disease. When gross anatomical changes in the bodily organs have once been produced it is hopeless to expect that remedial agents, whether old or new, will restore functional efficiency. But given the recognition of the first steps of disease there are many reasons for believing that these could be effectively countered by the administration of chemical or other agents, and that when the nature of these early changes is known appropriate remedies for them could be devised and prepared.

